# Supplementary material for: Spiritual Distress, Hopelessness, and Depression in Palliative Care: Simultaneous Concept Analysis
Source: Healthcare (Basel). 2024 May 7;12(10):960. doi: 10.3390/healthcare12100960 (PMC11121139; doi:10.3390/healthcare12100960)
Supplement: Supplementary file 1 [file healthcare-12-00960-s001.zip › healthcare-2944587-supplementary.pdf]

Supplementary Materials

Concept: Spiritual distress

Table S1. Search strategy for spiritual distress.

Database	Search strategy
**PubMed and Medline**	(“Spiritual distress” [tiab] OR “Existential distress” [tiab] OR “Lack of meaning” [tiab] OR “Suffering” [tiab] OR “Spiritual struggle” [tiab] OR “Spiritual crisis” [tiab] OR “Spiritual anguish” [tiab]) AND (“Palliative care” [MESH] OR “Terminal Care” [MESH] OR “End of life” [abstract] OR “Terminally ill” [tiab]) AND (“Caregivers” [MESH] OR “Carer*” [tiab] OR “Care Giver*” [tiab] OR “Spouse Caregiver*” [tiab] OR “Family Caregiver*” [tiab])
**CINAHL, MedicLatina, LILACS, and SciELO**	(“Spiritual distress” [abstract] OR “Existential distress” [abstract] OR “Lack of meaning” [abstract] OR “Suffering” [abstract] OR “Spiritual struggle” [abstract] OR “Spiritual crisis” [abstract] OR “Spiritual anguish” [abstract]) AND (“Palliative care” [abstract] OR “Terminal Care” [abstract] OR “End of life” [abstract] OR “Terminally ill” [abstract]) AND (“Caregivers” [abstract] OR “Carer*” [abstract] OR “Care Giver*” [abstract] OR “Spouse Caregiver*” [abstract] OR “Family Caregiver*” [abstract])

Concept: Hopelessness

Table S2. Search strategy for hopelessness.

Database	Search strategy
**PubMed and Medline**	(Hopelessness [tiab] OR Desperation [tiab] OR Desperateness[tiab] OR Despondency [tiab]) AND (“Palliative care” [MESH] OR “Terminal Care” [MESH] OR “End of life” [abstract] OR “Terminally ill” [tiab]) AND (“Caregivers” [MESH] OR “Carer*” [tiab] OR “Care Giver*” [tiab] OR “Spouse Caregiver*” [tiab] OR “Family Caregiver* ” [tiab])
**CINAHL, PsycINFO, MedicLatina, LILACS anf SciELO**	(Hopelessness [abstract] OR Desperation [abstract] OR Desperateness [abstract] OR Despondency [abstract]) AND (“Palliative care” [abstract] OR “Terminal Care” [abstract] OR “End of life” [abstract]OR “Terminally ill” [abstract]) AND (“Caregivers” [abstract] OR “Carer*” [abstract] OR “Care Giver*” [abstract] OR “Spouse Caregiver*” [abstract] OR “Family Caregiver*” [abstract])

Concept: Depression

Table S3. Search strategy for depression.

Database	Search strategy
**PubMed and Medline**	(Depression [MESH] OR Depressive Disorder [MESH] OR Sadness[tiab]) AND (“Palliative care” [MESH] OR “Terminal Care” [MESH] OR “End of life” [tiab]OR “Terminally ill” [tiab]) AND (“Caregivers” [MESH] OR “Carer*” [tiab] OR “Care Giver*” [tiab] OR “Spouse Caregiver*” [tiab] OR “Family Caregiver*” [tiab])
**CINAHL, MedicLatina, LILACS and SciELO**	(Depression* [abstract] OR Depressive Disorder [abstract] OR Sadness [abstract]) AND (“Palliative care” [abstract] OR “Terminal Care” [abstract] OR “End of life” [abstract] OR “Terminally ill” [abstract]) AND (“Caregivers” [abstract] OR “Carer*” [abstract] OR “Care Giver*” [abstract] OR “Spouse Caregiver*” [abstract] OR “Family Caregiver*” [abstract])

Selection process of spiritual distress

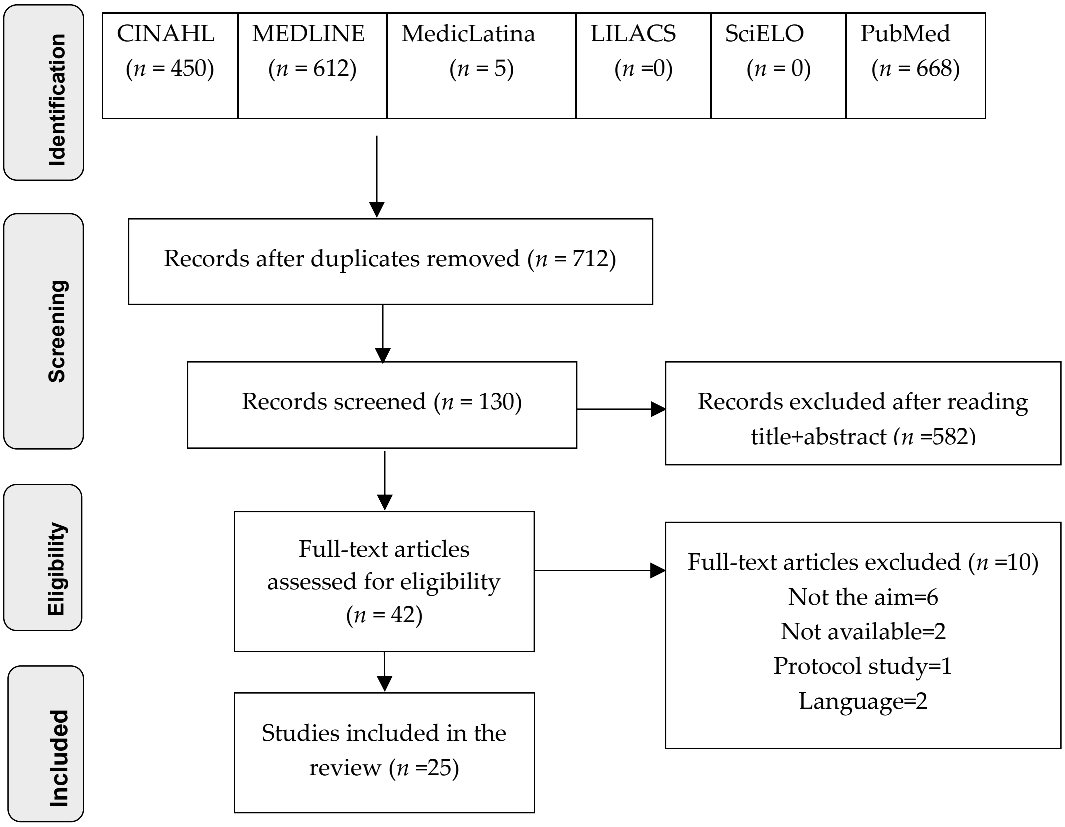

Figure S1. PRISMA flow diagram of the selection process of spiritual distress.

Selection process of hopelessness

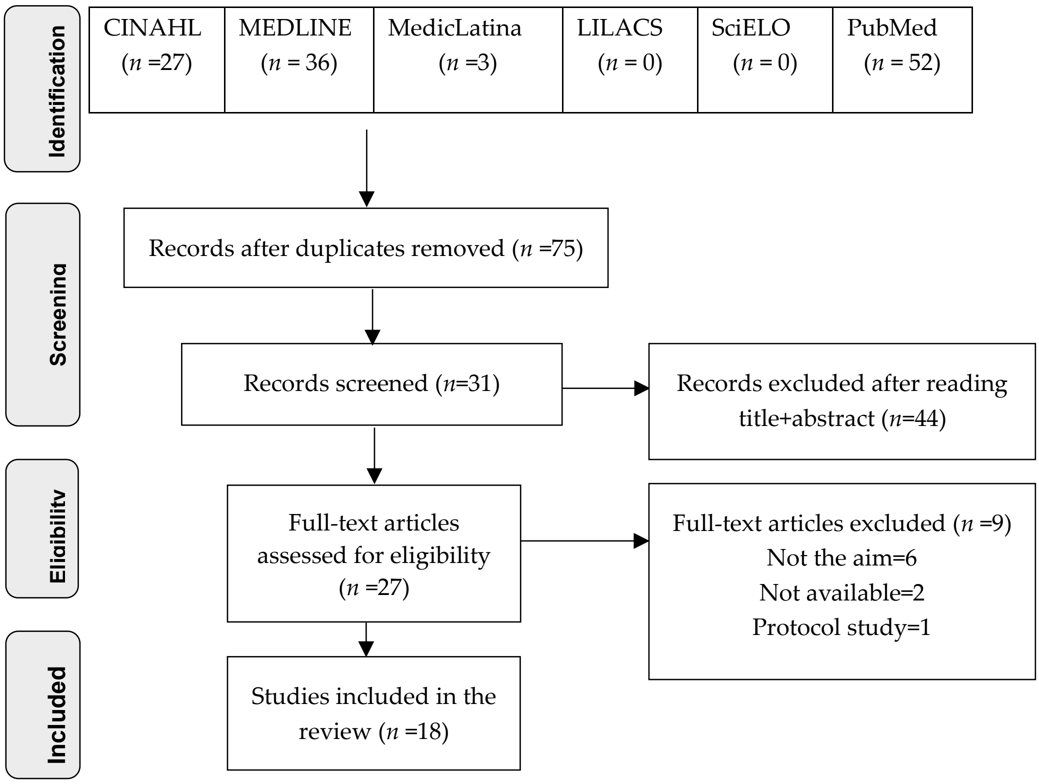

Figure S2. PRISMA flow diagram of the selection process of hopelessness.

Selection process of depression

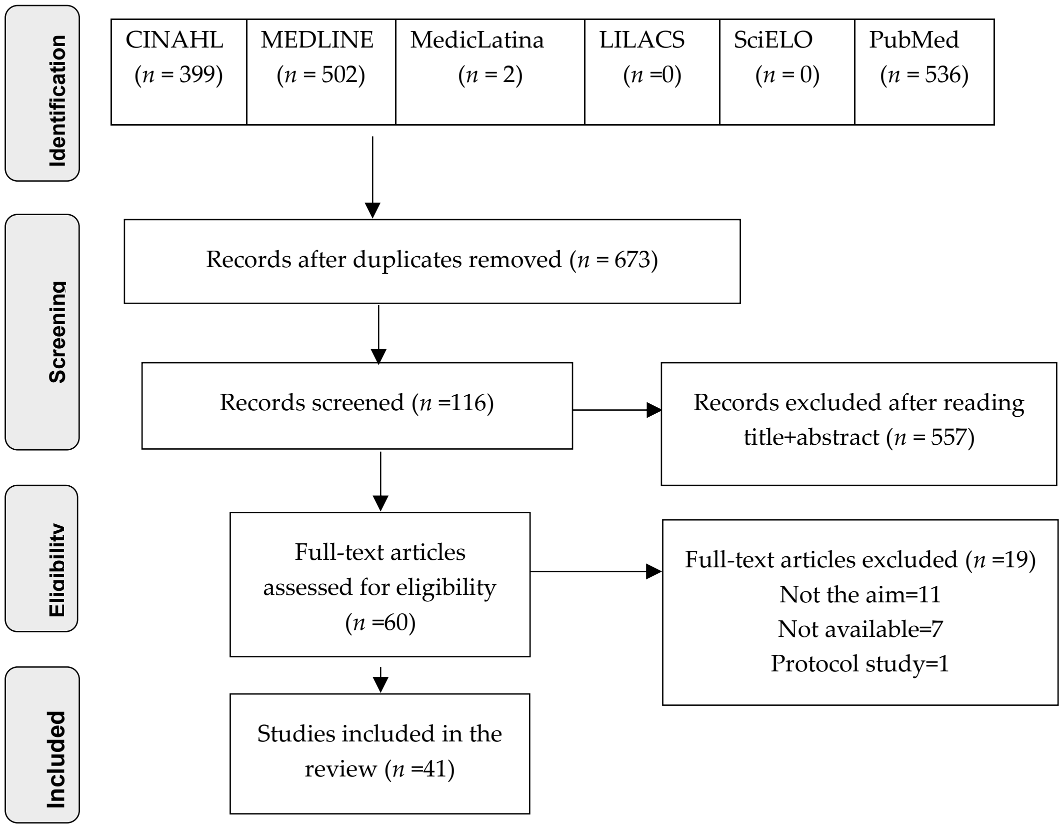

Figure S3. PRISMA flow diagram of the selection process of depression.

Table S4. Included studies descripition of spiritual distress.

Authors	Year	Journal	Aim	Methods
Abbas & Dein	2011	Mental Health, Religion & Culture	To investigate the difficulties that healthcare professionals encounter while Addressing the spiritual care needs of a patient.<br>To establish the views of these professionals concerning how they might deal with and learn to ask about their patients' spirituality	Qualitative methods. Focus group
Beng et al.	2014	American Journal of Hospice & Palliative Medicine	To explore the experiences of suffering caused by interactions with Health care providers in the hospital setting	Secondary analysis of two qualitative studies
Benites et al.	2022	Supportive Care in Cancer	To understand the spiritual and existential experience of family caregivers of patients with advanced cancer<br>facing the end of life in Brazil	Interpretative phenomenological analysis
Bhatnagar et al.	2017	Indian Journal of Palliative Care	To describe the most common signs of spiritual distress in Indian palliative care patients, assess differences between male and female participants, and formulate contextually appropriate recommendations for spiritual care based on this data	Quantitative and cross-sectional study
Boston et al.	2011	Journal of Pain and Symptom Management	To review the literature specifically related to existential suffering in palliative care in terms of the significance of existential suffering in end-of-life care, definitions, conceptual frameworks, and interventions	Systematic literature
Boston & Mount	2006	Journal of Pain and Symptom Management	To how palliative caregivers conceptualize, identify, and provide for spiritual and existential domains of care.	Qualitative study. Focus groups
Chan et al.	2016	SAGE Open Medical Case Reports	To assess the psycho-spiritual distress of an elderly with advanced congestive Heart failure	Case study
Delgado-Guay et al.	2016	Palliative & Supportive Care	To assess of spiritual distress/spiritual pain among patients in a supportive/palliative care clinic	Cross-sectional and correlational study
Delgado-Guay et al.	2013	American Journal of Hospice & Palliative Medicine	To determine the frequency of and associations among spirituality, religiosity, and spiritual pain.<br>To determine how these factors were associated with symptom expression and coping strategies	Cross-sectional Survey

Edwards et al.	2010	Palliative Medicine	To synthesize qualitative literature on spirituality and spiritual care at the end of life	Meta-study of Qualitative research
Foster et al.	2012	Journal of Hospice & Palliative Nursing	Highlights the nursing role in assessing and managing symptoms of spiritual suffering In children living with life-threatening conditions	Case study
Gielen et al.	2017	Journal Of Religion and Health	To describe the prevalence and nature of spiritual distress among Indian palliative care patient	Cross-sectional and correlational study
Hartogh	2017	Medicine, Health Care, and Philosophy	To conduct a theoretical work regarding suffering and dying well	Theoretical
Hui et al.	2011	The American Journal of Hospice & Palliative Care	To determine the frequency of spiritual distress and its relationship with physical and emotional distress	Cross-sectional and correlational study
Mako et al.	2006	Journal of Palliative Medicine	To explore the multidimensional nature of spiritual pain, in patients with end-stage cancer, in relation to physical pain, symptom severity, and emotional distress.	Mixed-methods
Meeker et al.	2014	Journal of Pain & Symptom Management	To investigate how patients with advanced illness and their primary caregivers experienced and responded to health care needs and decision making and how some dyads moved toward comfort-focused care	Qualitative study using the grounded theory method
Murray et al.	2007	Journal of Pain & Symptom Management	To identify and compare changes in the psychological, social, and spiritual needs of people with end-stage disease during their last year of life	Qualitative longitudinal research
Paal et al.	2020	Annals of Palliative Medicine	To reflect on the current state of art in spiritual care as an integrated palliative care approach for patients with neurodegenerative diseases and their caregivers	Literature review
Pessin et al.	2015	Current Opinion in Supportive and Palliative Care	To explore the existential concerns of professional caregivers and highlight their impact on psychosocial outcomes, such as burnout	Comprehensive literature review
Puchalski et al.	2004	Clinical Geriatric Medicine	To address Spirituality, religion, and healing in palliative care	Theoretical

Schultz et al.	2017	Journal of Pain and Symptom Management	To examine the extent to which this measure is identical to a variety of other measures, such as spiritual well-being, spiritual injury, spiritual pain, and general distress.	Cross-sectional and correlational study
Selman et al.	2007	Current Opinion in Supportive and Palliative Care	To informs clinicians of the importance of understanding, assessing and managing patients’ psychosocial and spiritual needs.	Theoretical
Siler et al.	2021	The American Journal of Hospice & Palliative Care	To understand older African Americans' perspectives on how spirituality influences chronic illness experiences to inform the development of a culturally tailored palliative care intervention.	Qualitative study. Focus groups
Velosa et al.	2017	Religions	To identify the prevalence and the clinical indicators of depression and spiritual distress in palliative patients in primary care	Quantitative, observational and cross-sectional study
Walbaum et al.	2024	Psychooncology	To synthesize the quantitative literature on existential distress among family caregivers of patients with advanced cancer, focusing on its prevalence, association with mental disorders, as well as with sociodemographic, disease, and treatment-related factors	Systematic review and meta-analysis

Table S5. Included studies descripition of hopelessness

Author	Year	Journal	Aim	Methods
Abdullah et al.	2020	Journal of Pain and Symptom Management	To review the preferences and experiences of muslim patients and their families in muslim-majority countries for end-of-life care	Systematic review
Boucher et al.	2018	Journal of Pain & Symptom Management	To understand opportunities for palliative care interventions in this population	Qualitatitive study
Breitbart et al.	2000	JAMA	To assess the prevalence of desire for hastened death among terminally ill cancer patients and to identify factors corresponding to desire for hastened death.	Prospective survey
Centers	2001	Journal of Palliative Care	To explore our "power to heal people and their lives", whether those people are the patient, the family, or the professional	Theoretical
Filibert et al.	2001	Journal of Pain & Symptom Management	To identify the possible vulnerability factors of suicide in five terminal cancer patients who committed suicide while they were cared for at home by well-trained palliative care teams, a psychological autopsy study was carried out by reviewing their medical records; their report of symptoms at the time of car	Qualitative study
Misko et al.	2015	Revista Latino-Americana De Enfermagem	To understand the family's experience of the child and/or teenager in palliative care and building a representative theoretical model of the process experienced by the family.	Qualitative study. Symbolic Interactionism as theoretical referential
Mystakidou et al.	2007	International Journal of Psychiatry in Medicine	To evaluate the prevalence of clinical characteristics and risk factors for hastened death in advanced cancer patients.	Quantitative and cross-sectional study
Mystakidou et al.	2009	Archives of Psychiatric Nursing	To assess the relationship of hopelessness, anxiety, distress, and preparatory grief, as well as their predictive power to hopelessness	Quantitative and cross-sectional study
Mystakidou et al.	2007	Cancer Nursing	To investigate whether the advanced cancer patients' caregivers' depression and	Quantitative and cross-sectional study

Mystakidou et al.	2008	Depress Anxiety	To evaluate the preparatory grief process in advanced cancer patients and its relationship with hopelessness, depression and an	Quantitative and cross-sectional study
Olsman et al.	2015	Palliative & Supportive Care	To describe hope, hopelessness, and despair over time, as experienced by palliative care patients	Qualitative longitudinal method based on narrative theories
Parpa et al.	2019	Supportive Care in Cancer	To investigate the relationship between hopelessness and desire for hastened death and if depression may be a moderator and/or mediator role in patients with advanced cancer.	Quantitative and cross-sectional study
Poppe	2020	Nursing Ethics	To conduct a conceptual consideration related to concepts of hope and its absence, hopelessness, are seen as crucial in palliative care for people with motor neurone disease	Theoretical
Rodin et al.	2009	Social Science & Medicine (1982)	To contribute regarding specific psychosocial and disease-related variables to the prediction of depression, hopelessness, and the desire for hastened death among terminally ill cancer patients	Quantitative and cross-sectional study
Rosenfeld et al.	2011	Psychological Assessment	To assessing hopelessness in terminally ill cancer patients	Qualitative study
Somasundaram et al.	2016	Indian Journal of Palliative Care	To examined the relationship between resilience, social support, and hopelessness among cancer patients treated with curative and palliative care.	Quantitative and cross-sectional study
van Laarhoven et al.	2011	Cancer Nursing	To assess coping strategies in curatively treated and palliative-cancer patients no longer receiving anticancer treatment and to examine the relation of these coping strategies with quality of life, depression, and hopelessness	Quantitative, descriptive and cross-sectional study
Zimmermann et al.	2016	Canadian Medical Association Journal	To examine perceptions of palliative care among patients with advanced cancer and their caregivers.	Grounded theory

Table S6. Included studies descripition of depression.

Authors	Year	Journal	Aim	Methods
Abreu & Junior	2018	Journal of Nursing UFPE / Revista de Enfermagem UFPE	To identify the repercussions of caring for a cancer patient in Palliative Care and the importance of nursing in supporting family caregivers.	Literature review
Azevedo et al.	2017	Revista da Escola de Enfermagem da USP	To analyze the relationship between social support, quality of life and depression in patients eligible for palliative care treated in Primary Health Care in a city in the interior of Minas Gerais, Brazil.	Cross-sectional and correlational study
Bekelman et al.	2008	International Journal of Cardiology	Our objective is to help define the role of palliative care in the treatment of older adults with heart failure, providing clinicians with strategies that acknowledge the complexity of older persons with heart failure and have the potential to improve outcomes for these patients	Theoretical
Block	2001	JAMA	To understand phycological concerns of pancreatic patients at end of life	Case study
Carter & Chang	2000	Cancer Nursing	To describe and explore the relation between caregiver sleep and depression.	Cross-sectional and correlational design
Chan et al..	2009	Social Workers Health Care	To explore the relationship of family-related factors and psychosocial outcomes among Hong Kong Chinese cancer patients in palliative care	Clinical data mining was adopted as the research method
Cheng et al.	1994	Psychological Reports	To identify the psychological needs in caregivers in terminally ill patient's	Cross-sectional and correlational design
Collins et al.	2020	Palliative Medicine	To describe the experience and support needs of caring for children with life-limiting conditions and examine the level of distress and quality-of-life experienced by parents.	Cross-sectional, prospective, quantitative study guided by an advisory group
Corà et al.	2012	Cancer Nursing	To examine psychological and cardiovascular responses in terminal cancer caregivers.	Longitudinal study, a paradigm of repeated measurements

Delalibera et al.	2018	Ciencia & Saude Coletiva	To characterize the family caregiver in palliative care, evaluating the circumstances and consequences of care and preparation for the loss of the loved one	Quantitative, prospective and longitudinal study.
Delalibera et al.	2015	Ciencia & Saude Coletiva	To conduct a systematic review of the literature on the burden of caregiving, related factors and the consequences for family caregivers of advanced stage cancer patients or patients in end-of-life or palliative care	Systematic review
Dipio et al.	2022	Palliative & supportive care	To determine prevalence and factors associated with depressive symptoms among family caregivers of palliative care patients at Hospice Africa Uganda	Mixed-methods
Fasse et al.	2015	Psychooncology	To describing depressive symptoms and depression among spouses who care for palliative cancer patients and to highlighting important factors explaining these symptoms	Survey
Fisher et al.	2014	BMC Palliative Care	To determine the prevalence of depressive symptoms and risk factors associated with them in a large sample of palliative home care patients.	Quantitative and cross-sectional study
Gonzalez et al.	2021	European Journal of Cancer Care	To examine how the burden of caregivers of patients with an advanced oncological illness mediates the relationship between positive aspects of care, depression and anxiety.	Quantitative study with a cross-sectional design
Govina et al.	2019	Asia-Pacific Journal of Oncology Nursing	To investigate the factors associated with family caregivers' anxiety and depression when caring for patients with advanced cancer in Greec	Cross-sectional, exploratory, and correlational study t
Haley et al.	2003	Journal of Palliative Medicine	To examines the applicability of a stress process model for spousal caregivers of the terminally ill	Quantitative and cross-sectional study
Hatano et al.	2022	Psycho-oncology	To determine association between experiences of advanced cancer patients at the end of life and depression in their bereaved caregivers	Quantitative and longitudinal study
Hirdes et al.	2012	Palliative & Supportive Care	To examine predictors of caregiver distress among community based palliative care clients	Quantitative and cross-sectional study
Jo et al.	2007	Palliative & Supportive Care	To examine the perspectives of both the spousal caregiver and care recipient on the caregiving experience in home-based palliative care	Qualitative study

Khalil et al.	2021	Japanese Psychological Research	To examine the effect of depressive and anxiety symptoms on physical and psychological quality of life in end-stage renal disease patient-family caregiver dyads using the Actor-Partner Interdependence Model	Quantitative, descriptive cross-sectional study
Kim et al.	2017	Korean Journal of Hospice & Palliative Care	To examine the associations between patient's symptoms themselves and family caregiver (FC)'s depression in the palliative phase	Quantitative and cross-sectional study
Kochuvilayil & Varma	2022	Journal of Palliative Care	To determine the factors associated with screening positive for depression among women caregivers of primary palliative care patients	Quantitative and cross-sectional study
Lai et al.	2018	European Journal of Cancer Care	To investigate the association between spirituality, burden, and psychological state in caregivers of terminally ill cancer patients	Quantitative and cross-sectional study
Macchi et al.	2020	Annals of Palliative Medicine	To understand patient and caregiver predictors of caregiver burden in Parkinson's disease from a palliative care approach.	Cross-sectional analysis and a randomized trial
Mystakidou et al.	2007	Cancer Nursing	To investigate whether the advanced cancer patients' caregivers' depression and hopelessness are affected by patients' demographic and clinical characteristics and by caregivers' sociodemographic variables	Quantitative and cross-sectional study
Nipp et al.	2016	Annals of Oncology: Official Journal of the European Society for Medical Oncology	To describe rates of depression and anxiety in family caregivers s of patients with incurable cancer and identify factors associated with these symptoms to determine those at greatest risk for psychological distress.	Cross-sectional analysis and a randomized trial
Olagunju et al.	2016	Annals of Palliative Medicine	To investigate if child's symptom burden is related to depressive symptoms in caregivers.	Quantitative, descriptive and cross-sectional study
Özdemir et al.	2020	Perspectives in Psychiatric Care	To examine the psychosocial problems and spiritual coping styles of the family caregivers related to patients receiving palliative care	Quantitative and cross-sectional study
Rabkin et al.	2009	Psycho-Oncology	To determine whether new-onset clinical depression emerges over time, and whether positive and negative mood levels change among patients with terminal cancer	Quantitative study with a cross-sectional design

Rhondali et al..	2015	Palliative Support Care	To examine caregivers' perceptions of depression among advanced cancer patients.	Grounded theory
Shaffer et al.	2017	Neurocrital Care	To compared anxiety and depressive symptomatology shortly following diagnosis among patients facing incurable cancer and their family caregivers	Quantitative study with a cross-sectional design
Sherif et al.	2001	Eastern Mediterranean Health Journal	To compare anxiety, depression and caregiver's´ quality of life in oncology and chronically ill patients	Quantitative study with a cross-sectional design
Siminoff et al.	2010	Psychooncology	To investigate depressive symptomatology in lung cancer patients and their identified caregiver.	Quantitative study with a cross-sectional design
Smith et al.	2003	Palliative Medicine	To investigate the hypothesis that illness severity, pain, anxiety and depression are all associated with impaired health-related quality of life a	Quantitative study with a cross-sectional design
Tay et al.	2022	Psycho-Oncology	To examine the association between contextual characteristics and appraisal factors on family caregivers' mental health and well-being.	Quantitative study with a cross-sectional design
Tang et al.	2013	Psychooncology	To identify trajectories of depressive symptoms among caregivers providing end-of-life care to cancer patients and profiled the unique characteristics of caregivers within each trajectory	Quantitative study with a longitudinal design
Tang et al.	2007	Palliative Medicine	To identify those family caregivers of Taiwanese terminally ill cancer patients who are at risk of experiencing depressive distress from the following three categories of predisposing facto	Quantitative study with a cross-sectional design
Tanriverdi et al.	2016	European Journal Cancer Care	To evaluate the burden associated with the personal, social and financial responsibilities assumed by caregivers of cancer patients and to determine the prevalence of depression and depressionrelated variables among caregivers of cancer patients who are receiving chemotherapy in Turkey	Quantitative study with a cross-sectional design
Valeberg & Grov	2013	European Journal of Oncology Nursing	To examine the level of symptom burden in a sample of cancer patients in a curative and palliative phase	Quantitative, descriptive and cross-sectional study

Wasner et al.	2013	Journal of Social Work in End-of-Life & Palliative Care	To examine the caregivers' personal experiences, quality of life, burden of care, and psychological well-being	Quantitative study with a cross-sectional design

Table S7. Process model of spiritual distress with references of included studies.

Antecedents	References
**Awareness of terminality death**	Beng et al. (2014); Benites et al. (2022); Delgado-Guay et al. (2013); Foster et al. (2012); Hartogh (2017); Meeker et al. (2014); Murray et al. (2007); Pessin et al. (2015); Schultz et al. (2017);
**Caregivers burden**	Benites et al. (2022); Boston et al. (2011); Hartogh (2017); Murray et al. (2007); Pessin et al. (2015); Selman et al. (2007);
**Existential issues**	Benites et al. (2022); Bhatnagar et al. (2017); Boston et al. (2011); Boston &Mount (2006); Chan et al. (2016); Delgado-Guay et al. (2013); Foster et al. (2012); Gielen et al. (2017); Hartogh (2017); Hui et al. (2011); Murray et al. (2007); Pessin et al. (2015); Puchalski et al. (2004); Schultz et al. (2017); Selman et al. (2007); Siler et al. (2021); Walbaum et al. (2024);
**Lack of financial support**	Delgado-Guay et al. (2016); Edwards et al. (2010);
**Lack of social support**	Selman et al. (2007); Murray et al. (2007); Selman et al. (2007);
**Loss of autonomy**	Edwards et al. (2010); Hartogh (2017); Murray et al. (2007);
**Loss of control**	Edwards et al. (2010); Hartogh (2017);
**Loss relationship**	Edwards et al. (2010); Murray et al. (2007);
**Rupture of belief system or person's spiritual/religious orienting system and/or their belief's**	Abbas & Dein (2011); Bhatnagar et al. (2017); Gielen et al. (2017); Hartogh (2017); Mako et al. (2006); Murray et al. (2007); Puchalski et al. (2004); Siler et al. (2021);
**Sense of disintegration of the self**	Boston & Mount (2006); Edwards et al. (2010); Mako et al. (2006); Murray et al. (2007); Pessin et al. (2015); Puchalski et al. (2004); Walbaum et al. (2024);
**Traumatic life events**	Hui et al. (2011); Velosa et al. (2017);
**Uncertainty about future**	Benites et al. (2022); Bhatnagar et al. (2017); Boston et al. (2011); Gielen et al. (2017); Murray et al. (2007); Puchalski et al. (2004); Schultz et al. (2017);
**Unmet spiritual needs**	Abbas & Dein (2011); Beng et al. (2014); Delgado-Guay et al. (2013); Edwards et al. (2010); Mako et al. (2006); Paal et al. (2020); Siler et al. (2021);
**Attributes**	**References**
**Alienation**	Hui et al. (2011); Mako et al. (2006);
**Disconnection from self, from others, and from God or the transcendent**	Benites et al. (2022); Gielen et al. (2017); Mako et al. (2006); Murray et al. (2007); Pessin et al. (2015); Siler et al. (2021);
**Existential issues, frequents thought about death**	Bhatnagar et al. (2017); Gielen et al. (2017); Hartogh (2017); Mako et al. (2006); Murray et al. (2007); Pessin et al. (2015); Puchalski et al. (2004); Schultz et al. (2017); Selman et al. (2007); Siler et al. (2021); Walbaum et al. (2024);
**Fear for the future**	Bhatnagar et al. (2017); Edwards et al. (2010); Gielen et al. (2017); Hartogh (2017); Mako et al. (2006); Murray et al. (2007); Siler et al. (2021);
**Feeling abandoned by God, loss of faith and/or a religious/spiritual belief**	Bhatnagar et al. (2017); Benites et al. (2022); Gielen et al. (2017); Mako et al. (2006); Murray et al. (2007); Paal et al. (2020); Siler et al. (2021);
**Feel anger and punished by God**	Bhatnagar et al. (2017); Edwards et al. (2010); Gielen et al. (2017); Murray et al. (2007); Siler et al. (2021);
**Guilt**	Benites et al. (2022); Bhatnagar et al. (2017); Edwards et al. (2010); Gielen et al. (2017); Hartogh (2017); Hui et al. (2011);
**Inability to self- forgiveness**	Gielen et al. (2017).
**Isolation**	Mako et al. (2006); Murray et al. (2007); Puchalski et al. (2004);
**Loneliness**	Benites et al. (2022); Bhatnagar et al. (2017); Gielen et al. (2017); Walbaum et al. (2024);

**Loss of meaning and purpose in life**	Bhatnagar et al. (2017); Hartogh (2017); Hui et al. (2011); Murray et al. (2007); Puchalski et al. (2004); Schultz et al. (2017); Selman et al. (2007);
**Loss or altered sense of self**	Mako et al. (2006); Murray et al. (2007); Puchalski et al. (2004);
**Not feel at peace**	Boston et al. (2011); Delgado-Guay et al. (2013); Edwards et al. (2010) Gielen et al. (2017); Schultz et al. (2017);
**Questioning the meaning of their experiences**	Bhatnagar et al. (2017); Hartogh (2017); Hui et al. (2011); Murray et al. (2007); Selman et al. (2007);
**Suffering**	Benites et al. (2022); Bhatnagar et al. (2017); Gielen et al. (2017); Hartogh (2017); Murray et al. (2007); Puchalski et al. (2004); Schultz et al. (2017);
**Outcomes**	**References**
**Anxiety**	Delgado-Guay et al. (2013); Mako et al. (2006); Pessin et al. (2015); Walbaum et al. (2024);
**Decrease of quality-of-life**	Boston et al. (2011); Delgado-Guay et al. (2013); Murray et al. (2007);
**Decrease spiritual well-being**	Murray et al. (2007);
**Decrease general well-being**	Siler et al. (2021);
**Denial**	Delgado-Guay et al. (2013);
**Depression**	Boston et al. (2011); Chan et al. (2016); Hui et al. (2011); Mako et al. (2006); Velosa et al. (2017);
**Hopelessness**	Benites et al. (2022); Edwards et al. (2010); Hartogh (2017); Hui et al. (2011); Murray et al. (2007); Puchalski et al. (2004); Walbaum et al. (2024);
**More behavioral disengagement**	Delgado-Guay et al. (2013);
**More dysfunctional coping strategies**	Delgado-Guay et al. (2013);
**More prone to severe /increase pain**	Gielen et al. (2017); Hui et al. (2011).

References

1. Abbas, S.Q.; Dein, S. The difficulties assessing spiritual distress in palliative care patients: a qualitative study. *Mental Health, Religion & Culture* **2011**, *14*, 341–352. https://doi.org/10.1080/13674671003716780
2. Beng, T.S.; Guan, N. C.; Jane, L. E.;Chin, L. E. health care interactional suffering in palliative care. *AJHPM***2014**, *31*, 307–314. https://doi.org/10.1177/1049909113490065
3. Benites, A. C.; Rodin, G.; de Oliveira-Cardoso, É. A.; Dos Santos, M. A. "You begin to give more value in life, in minutes, in seconds": spiritual and existential experiences of family caregivers of patients with advanced cancer receiving end-of-life care in Brazil. Support Care Cancer **2022**, 30, 2631–2638. https://doi.org/10.1007/s00520-021-06712-w
4. Bhatnagar, S.; Gielen, J.; Satija, A.; Singh, S. P.; Noble, S.; Chaturvedi, S. K. Signs of spiritual distress and its implications for practice in indian palliative care. *Indian Jounal of Palliative Care* **2017,** *23*, 306-311. https://doi.org/10.4103/IJPC.IJPC_24_17
5. Boston, P.; Bruce, A.; Schreiber, R. Existential suffering in the palliative care setting: an integrated literature review. *J Pain Symptom Manag* **2011**, *41*, 604–618. https://doi.org/10.1016/j.jpainsymman.2010.05.010
6. Boston, P. H.; Mount, B. M. The caregiver's perspective on existential and spiritual distress in palliative care. *J Pain Symptom Manag* **2006**, *32*, 13–26. https://doi.org/10.1016/j.jpainsymman.2006.01.009
7. Chan, K.Y.; Lau, V. W.K.; Cheung, K.C.; Chang, R. S.K.; Chan, M.L. Reduction of psycho-spiritual distress of an elderly with advanced congestive heart failure by life review interview in a palliative care day center. *SAGE Open Medical Case Reports* **2016**, *4*, 2050313X16665998. https://doi.org/10.1177/2050313X16665998
8. Delgado-Guay, M. O.; Chisholm, G.; Williams, J.; Frisbee-Hume, S., Ferguson, A. O.; Bruera, E. Frequency, intensity, and correlates of spiritual pain in advanced cancer patients assessed in a supportive/palliative care clinic. *Palliat Support Care* **2016**, *14*, 341–348. https://doi.org/10.1017/S147895151500108X
9. Delgado-Guay, M. O.; Parsons, H. A.; Hui, D., Cruz; M. G. D. la, Thorney, S.; Bruera, E. Spirituality, religiosity, and spiritual pain among caregivers of patients with advanced cancer. *AJHPM* **2013**, *30*, 455–461. https://doi.org/10.1177/1049909112458030

10. Edwards, A.; Pang, N.; Shiu, V.; Chan, C. Review. The understanding of spirituality and the potential role of spiritual care in end-of-life and palliative care: a meta-study of qualitative research. *Palliat Med* **2010**, *24*, 753–770. https://doi.org/10.1177/0269216310375860
11. Foster, T. L.; Bell, C. J.; Gilmer, M. J. Symptom management of spiritual suffering in pediatric palliative care. *JHPN* **2012**, *14*, 109–117. https://doi.org/10.1097/njh.0b013e3182491f4b
12. Gielen, J.; Bhatnagar, S.; Chaturvedi, S. K. (2017). Prevalence and nature of spiritual distress among palliative care patients in india. *J Relig Health*, *56*(2), 530–544. https://doi.org/10.1007/s10943-016-0252-5
13. Hartogh, G. den. Suffering and dying well: on the proper aim of palliative care. *Medicine, Health Care, and Philosophy* **2017**, *20*, 413–424. https://doi.org/10.1007/s11019-017-9764-3
14. Hui, D.; de la Cruz, M.; Thorney, S.; Parsons, H. A.; Delgado-Guay, M.; Bruera, E. The Frequency and correlates of spiritual distress among patients with advanced cancer admitted to an acute palliative care unit. *AJHPM* **2011**, *28*, 264–270. https://doi.org/10.1177/1049909110385917
15. Mako, C.; Galek, K.; Poppito, S.R. Spiritual pain among patients with advanced cancer in palliative care. *J Palliat Med* **2006**, *9*, 1106-13. https://doi.org/10.1089/jpm.2006.9.1106
16. Meeker, M. A.; Waldrop, D. P.; Schneider, J.; Case, A. A. Contending with advanced illness: patient and caregiver perspectives. *J Pain Symptom Manag* **2014**, *47*, 887–895. https://doi.org/10.1016/j.jpainsymman.2013.06.009
17. Murray, S. A.; Kendall, M.; Grant, E.; Boyd, K.; Barclay, S.; Sheikh, A. Patterns of social, psychological, and spiritual decline toward the end of life in lung cancer and heart failure. *J Pain Symptom Manag* **2007**, *34*, 393–402. https://doi.org/10.1016/j.jpainsymman.2006.12.009
18. Paal, P.; Lex, K.M., Brandstötter,. C.; Weck, C.; Lorenzl, S. Spiritual care as an integrated approach to palliative care for patients with neurodegenerative diseases and their caregivers: a literature review. *Ann Palliat Med* **2020**, *9*, 2303-2313. https://doi.org/ 10.21037/apm.2020.03.37
19. Pessin, H.; Fenn, N.; Hendriksen, E.; DeRosa, A. P.; Applebaum, A. Existential distress among healthcare providers caring for patients at the end of life. *Curr Opin Support Palliat Care* **2015**, *9*, 77–86. https://doi.org/10.1097/SPC.0000000000000116
20. Puchalski, C. M.; Dorff, R. E.; Hendi, I. Y. Spirituality, religion, and healing in palliative care. *Clin Geriatr Med* **2004**, *20*, 689–714.
21. Schultz, M.; Meged-Book, T.; Mashiach, T.; Bar-Sela, G. Distinguishing between spiritual distress, general distress, spiritual well-being, and spiritual pain among cancer patients during oncology treatment. *J Pain Symptom Manag* **2017**, *54*, 66–73. https://doi.org/10.1016/j.jpainsymman.2017.03.018
22. Selman, L.; Beynon, T.; Higginson, I.J.; Harding, R. Psychological, social and spiritual distress at the end of life in heart failure patients. *Curr Opin Support Palliat Care* **2007**,*1*, 260-266. https://doi.org/10.1097/SPC.0b013e3282f283a3
23. Siler, S.; Arora, K.; Doyon, K.; Fischer, S. M. Spirituality and the illness experience: perspectives of african american older adults. *AJHPM* **2021**, *38*, 618–625. https://doi.org/10.1177/1049909120988280
24. Velosa, T.; Caldeira, S.; Capelas, M. L. Depression and spiritual distress in adult palliative patients: a cross-sectional study. *Relig* **2017**, *8*,156. https://doi.org/10.3390/rel8080156
25. Walbaum C; Philipp R; Oechsle K; Ullrich A; Vehling S. Existential distress among family caregivers of patients with advanced cancer: A systematic review and meta-analysis. *Psychooncology* **2024**, *33*, e6239. . https://doi.org/10.1002/pon.6239

Table S8. Process model of hopelessness with references of included studies.

Antecedents	References
**Caregiver burden and sense of being a burden by the patients**	Boucher et al. (2018); Filiberti et al. (2001); Mystakidou et al. (2007b); Parpa et al. (2019); Rodin et al. (2009); Zimmermann et al. (2016);
**Impaired relation between caregiver and patient**	Mystakidou et al. (2007b);
**Impaired medical curative treatment**	Boucher et al. (2018); Filiberti et al. (2001); Olsman et al. (2015); Rosenfeld et al. (2011); Somasundaram & Devamani (2016); Zimmermann et al. (2016);
**Perception of an incurable/life-threatening illness**	Boucher et al. (2018); Breitbart et al. (2000); Centers (2001); Mystakidou et al. (2007); Mystakidou et al. (2007b); Olsman et al. (2015); Parpa et al. (2019); Poppe (2020); Rodin et al. (2009); Rosenfeld et al. (2011); Somasundaram & Devamani (2016); van Laarhoven et al. (2011); Zimmermann et al. (2016); Abdullah et al. (2020);
**Perception of a negative health condition**	Boucher et al. (2018); Centers (2001); Mystakidou et al. (2007b); Mystakidou et al. (2008); Olsman et al. (2015); Parpa et al. (2019); Poppe (2020); Rosenfeld et al. (2011); Somasundaram & Devamani (2016); van Laarhoven et al. (2011); Zimmermann et al. (2016);
**Physical and psychological deterioration**	Filiberti et al. (2001); Mystakidou et al. (2007a); Mystakidou et al. (2008); Parpa et al. (2019); Rodin et al. (2009); Zimmermann et al. (2016);
**Social isolation**	Boucher et al. (2018); Centers (2001);
**Attributes**	**References**
**Having given up on life**	Mystakidou et al. (2007b); Olsman et al. (2015); Rosenfeld et al. (2011); van Laarhoven et al. (2011);
**Inability to improve and control one's situation**	Boucher et al. (2018); Centers (2001); Filiberti et al. (2001); Olsman et al. (2015); Rosenfeld et al. (2011); Zimmermann et al. (2016);
**Lack of future expectations**	Boucher et al. (2018); Mystakidou et al. (2007b); Mystakidou et al. (2008); Parpa et al. (2019); Olsman et al. (2015); Rosenfeld et al. (2011); van Laarhoven et al. (2011);
**Lack of hope**	Abdullah et al. (2020); Breitbart et al. (2000); Centers (2001); Mystakidou et al. (2007b); Mystakidou et al. (2008); Poppe (2020); Rodin et al. (2009); Rosenfeld et al. (2011); Somasundaram & Devamani (2016); van Laarhoven et al. (2011); Zimmermann et al. (2016);
**Negative expectations about the future**	Boucher et al. (2018); Mystakidou et al. (2007b); Mystakidou et al. (2008); Parpa et al. (2019); Poppe (2020); Rodin et al. (2009); Rosenfeld et al. (2011); Somasundaram & Devamani (2016); van Laarhoven et al. (2011); Zimmermann et al. (2016);
**Negative feelings towards the future**	Boucher et al. (2018); Centers (2001); Mystakidou et al. (2008); Olsman et al. (2015); Parpa et al. (2019); Rosenfeld et al. (2011); Zimmermann et al. (2016);
**Negative thoughts and feelings**	Breitbart et al. (2000); Breitbart et al. (2000); Centers (2001); Mystakidou et al. (2007a); Mystakidou et al. (2008); Olsman et al. (2015); Parpa et al. (2019); Poppe (2020); Rodin et al. (2009); Rosenfeld et al. (2011); Somasundaram & Devamani (2016); Zimmermann et al. (2016);
**Sense of futility of life**	Rosenfeld et al. (2011);
**Uncertainty of the future**	Abdullah et al. (2020); Boucher et al. (2018); Mystakidou et al. (2008); Olsman et al. (2015); Parpa et al. (2019); Rosenfeld et al. (2021); Zimmermann et al. (2016); Abdullah et al. (2020);
**Outcomes**	**References**
**Depression**	Boucher et al. (2018); Breitbart et al. (2000); Filiberti et al. (2001); Mystakidou et al. (2007b); Mystakidou et al. (2008); Mystakidou et al. (2009); Olsman et al. (2015); Parpa et al. (2019); Rosenfeld et al. (2021); van Laarhoven et al. (2011);

**Despair**	Centers (2001); Olsman et al. (2015); Zimmermann et al. (2016);
**Fatality**	Centers (2001); Mystakidou et al. (2007a); Mystakidou et al. (2009); Olsman et al. (2015); Rodin et al. (2009); Zimmermann et al. (2016);
**Hastened death**	Breitbart et al. (2000); Parpa et al. (2019); Rosenfeld et al. (2021); van Laarhoven et al. (2011);
**Impaired quality of life**	Mystakidou et al. (2007a); Mystakidou et al. (2008); Poppe (2020);
**Suicidal ideation**	Breitbart et al. (2000); Filiberti et al. (2001); Rosenfeld et al. (2021).

References

1. Abdullah, R.; Guo, P.; Harding, R. Preferences and experiences of muslim patients and their families in muslim-majority countries for end-of-life care: A Systematic review and thematic analysis. *J Pain Symptom Manag* **2020**, *60*, 1223. https://doi.org/10.1016/j.jpainsymman.2020.06.032
2. Boucher, N. A.; Johnson, K. S.; LeBlanc, T. W. Acute leukemia patients' needs: qualitative findings and opportunities for early palliative care. *J Pain Symptom Manag* **2018**, *55*, 433–439. https://doi.org/10.1016/j.jpainsymman.2017.09.014
3. Breitbart, W.; Rosenfeld, B.; Pessin, H.; Kaim, M.; Funesti-Esch, J.; Galietta, M.; Nelson, C. J; Brescia, R. Depression, hopelessness, and desire for hastened death in terminally ill patients with cancer. *JAMA* **2000**, *284*, 2907–2911. https://doi.org/10.1001/jama.284.22.2907
4. Centers, L.C. Forum. Beyond denial and despair: ALS and our heroic potential for hope. *J Palliat Med* **2001**, *17*, 259–264.
5. Filiberti, A.; Ripamonti, C.; Totis, A.; Ventafridda, V.; De Conno, F., Contiero, P.; Tamburini, M.; Filiberti, A.; Ripamonti, C.; Totis, A.; Ventafridda, V.; De Conno, F.; Contiero, P; Tamburini, M. Characteristics of terminal cancer patients who committed suicide during a home palliative care program. *J Pain Symptom Manag* **2001**, *22*, 544–553.
6. Misko, M. D.; dos Santos, M. R.; Ichikawa, C. R. de F.; de Lima, R. A. G.; Bousso, R. S. The family's experience of the child and/or teenager in palliative care: fluctuating between hope and hopelessness in a world changed by losses. *Revista Latino-Americana de Enfermagem* **2015**, *23*, 560–567. https://doi.org/10.1590/0104-1169.0468.2588
7. Mystakidou, K.; Parpa, E.; Tsilika, E.; Pathiaki, M.; Galanos, A.; Vlahos, L. Depression, hopelessness, and sleep in cancer patients' desire for death. *Int J Psychiatryin Medicine* **2007**, *37*, 201–211. https://doi.org/10.2190/0509-7332-388n-566w
8. Mystakidou, K.; Tsilika, E.; Parpa, E.; Athanasouli, P.; Galanos, A.; Pagoropoulou, A.; Vlahos, L. Illness-related hopelessness in advanced cancer: influence of anxiety, depression, and preparatory grief. *Arch Psychi Nurs* **2009**, *23*(2), 138–147. https://doi.org/10.1016/j.apnu.2008.04.008
9. Mystakidou K; Tsilika E; Parpa E; Galanos A; Vlahos L. Caregivers of advanced cancer patients: feelings of hopelessness and depression. *Cancer Nurs* **2007**, *30*, 412–418. https://doi.org/10.1097/01.ncc.0000290807.84076.73
10. Mystakidou, K.; Tsilika, E.; Parpa, E.; Pathiaki, M.; Galanos, A.; Vlahos, L. The relationship between quality of life and levels of hopelessness and depression in palliative care. *Depression and Anxiety* **2008**, *25*, 730–736. https://doi.org/10.1002/da.20319
11. Olsman, E.; Leget, C.; Duggleby, W.; Willems, D. (2015). A singing choir: Understanding the dynamics of hope, hopelessness, and despair in palliative care patients. A longitudinal qualitative study. *Palliat Support Care* **2008**, *13*, 1643–1650. https://doi.org/10.1017/S147895151500019X
12. Parpa, E.; Tsilika, E.; Galanos, A.; Nikoloudi, M.; Mystakidou, K. Depression as mediator and or moderator on the relationship between hopelessness and patients' desire for hastened death. *Support Care Cancer* **2019**, *27*, 4353–4358. https://doi.org/10.1007/s00520-019-04715-2
13. Poppe, C. Hopelessness in palliative care for people with motor neurone disease: Conceptual considerations. *Nurs Ethics* **2020**, *27*, 316–320. https://doi.org/10.1177/0969733019901225
14. Rodin, G.; Lo, C.; Mikulincer, M.; Donner, A.; Gagliese, L.; Zimmermann, C. Pathways to distress: the multiple determinants of depression, hopelessness, and the desire for hastened death in metastatic cancer patients. *Soc Sci Med* **2009**, *68*, 562–569. https://doi.org/10.1016/j.socscimed.2008.10.037
15. Rosenfeld, B.; Pessin, H.; Lewis, C.; Abbey, J.; Olden, M.; Sachs, E.; Amakawa, L.; Kolva, E.; Brescia, R.; Breitbart, W. Assessing hopelessness in terminally ill cancer patients: Development of the hopelessness assessment in illness questionnaire. *Psychological Assessment* **2011**, *23*, 325–336. https://doi.org/10.1037/a0021767

16. Somasundaram, R. O.; Devamani, K. A. A comparative study on resilience, perceived social support and hopelessness among cancer patients treated with curative and palliative care. *Indian J Palliat Med* **2016**, *22*, 135–140. https://doi.org/10.4103/0973-1075.179606
17. van Laarhoven, H. W. M.; Schilderman, J.; Bleijenberg; G.; Donders, R.; Vissers, K. C.; Verhagen, C. A.; Prins, J. B. Coping, quality of life, depression, and hopelessness in cancer patients in a curative and palliative, end-of-life care setting. *Cancer Nurs* **2011**, *34*, 302–314. https://doi.org/10.1097/NCC.0b013e3181f9a040
18. Zimmermann, C.; Swami, N.; Krzyzanowska, M.; Leighl, N.; Rydall, A.; Rodin, G.; Tannock, I.; Hannon, B. Perceptions of palliative care among patients with advanced cancer and their caregivers. *CMAJ* **2016**, *188*, E217–E227. https://doi.org/10.1503/cmaj.151171

Table S9. Process model of depression with references of included studies.

Antecedents	References
**Caregiver burden and sense of being a burden by the patients**	Abreu & Júnior (2018); Delalibera et al. (2018); Delalibera et al. (2015); Dipio et al. (2022); Fisher et al. (2014); Gonzalez et al. (2021); Govina et al. (2019); Haley et al. (2003); Hatano et al. (2022); Hirdes et al. (2012); Kim et al. (2017); Kochuvilayil & Varma (2022); Jo et al. (2007); Lai et al. (2018); Macchi et al. (2020); Mystakidou et al. (2007); Nipp et al. (2016); Olagunju et al. (2016); Özdemir et al. (2020); Rabkin et al. (2009); Rhondali et al. (2015); Tang et al. (2013); Tang et al. (2007); Tanriverdi et al. (2016); Valeberg & Grov (2013); Wasner et al. (2013);
**Deterioration of health of the patients**	Carter & Chang (2000); Delalibera et al. (2015); Kim et al. (2017); Kochuvilayil & Varma (2022); Lai et al. (2018); Collins et al. (2019); Mystakidou et al. (2007); Özdemir et al. (2020); Rhondali et al. (2015); Tang et al. (2013); Valeberg & Grov (2013);
**Emotional exhaustion**	Cheng et al. (2009); Dipio et al. (2022); Mannarino (2020); Haley et al. (2003); Kim et al. (2017); Jo et al. (2007); Lai et al. (2018); Collins et al. (2019); Mystakidou et al. (2007); Rabkin et al. (2009);
**Impaired relation between caregiver and patient**	Carter & Chang (2000); Fasse et al. (2015); Haley et al. (2003); Jo et al. (2007); Mystakidou et al. (2007); Özdemir et al. (2020); Siminoff et al. (2010); Tang et al. (2013);
**Lack of financial support**	Abreu & Júnior (2018); Dipio et al. (2022); Kim et al. (2017); Kochuvilayil & Varma (2022); Jo et al. (2007); Özdemir et al. (2020); Smith et al. (2003); Tay et al. (2022); Wasner et al. (2013);
**Lack of social support**	Abreu & Júnior (2018); Azevedo et al. (2017); Cheng et al. (2009); Delalibera et al. (2018); Dipio et al. (2022); Haley et al. (2003); Hatano et al. (2022); Kochuvilayil & Varma (2022); Lai et al. (2018); Nipp et al. (2016); Özdemir et al. (2020); Tay et al. (2022); Tang et al. (2013); Wasner et al. (2013);
**Perception of an incurable/life-threatening illness**	Carter & Chang (2000); Delalibera et al. (2015); Fisher et al. (2014); Fisher et al. (2014); Kim et al. (2017); Collins et al. (2019); Mystakidou et al. (2007); Özdemir et al. (2020); Rabkin et al. (2009); Rhondali et al. (2015); Shaffer et al. (2017); Sherif et al. (2001); Tang et al. (2013); Tanriverdi et al. (2016); Valeberg & Grov (2013);
**Poor control of pain and symptoms**	Azevedo et al. (2017); Block (2001); Chan et al. (2009); Fisher et al. (2014); Collins et al. (2019); Olagunju et al. (2016); Özdemir et al. (2020); Rhondali et al. (2015); Sherif et al. (2001); Smith et al. (2003); Tang et al. (2013); Valeberg & Grov (2013);
**Psychological distress**	Chan et al. (2009); Cheng et al. (2009); Corà et al. (2012); Delalibera et al. (2015); Mannarino (2020); Fisher et al. (2014); Govina et al. (2019); Hirdes et al. (2012); Lai et al. (2018); Collins et al. (2019); Mystakidou et al. (2007); Nipp et al. (2016); Olagunju et al. (2016); Özdemir et al. (2020); Rabkin et al. (2009); Shaffer et al. (2017); Tang et al. (2013); Tang et al. (2007); Tanriverdi et al. (2016);
**Stress**	Abreu & Júnior (2018); Block (2001); Carter & Chang (2000); Corà et al. (2012); Govina et al. (2019); Haley et al. (2003); Haley et al. (2003); Hirdes et al. (2012); Sherif et al. (2001); Siminoff et al. (2010);
**Attributes**	**References**
**Guilt**	Block (2001); Cheng et al. (2009); Rhondali et al. (2015); Sherif et al. (2001); Siminoff et al. (2010);
**Hopelessness**	Block (2001); Fisher et al. (2014); Mystakidou et al. (2007); Rhondali et al. (2015);
**Impaired self-esteem**	Block (2001); Fasse et al. (2015); Mystakidou et al. (2007); Rabkin et al. (2009);

**Impairment emotion processing**	Abreu & Júnior (2018); Block (2001); Corà et al. (2012); Delalibera et al. (2015); Fisher et al. (2014); Govina et al. (2019); Jo et al. (2007); Khalil et al. (2021); Macchi et al. (2020); Mannarino (2020); Collins et al. (2019); Nipp et al. (2016); Özdemir et al. (2020); Rabkin et al. (2009); Rhondali et al. (2015); Shaffer et al. (2017); Sherif et al. (2001); Siminoff et al. (2010); Smith et al. (2003); Tang et al. (2007); Tanriverdi et al. (2016); Valeberg & Grov (2013); Wasner et al. (2013);
**Impairment of cognitive functioning**	Block (2001);Bekelman et al. (2008); Carter & Chang (2000); Fisher et al. (2014); Hirdes et al. (2012); Mystakidou et al. (2007); Özdemir et al. (2020); Rhondali et al. (2015); Smith et al. (2003);
**Impairment of social functioning**	Cheng et al. (2009); Özdemir et al. (2020); Rabkin et al. (2009); Rhondali et al. (2015); Smith et al. (2003);
**No sense of a positive future**	Block (2001);
**Physical symptoms (fatigue, insomnia, lack of energy, etc);**	Block (2001); Bekelman et al. (2008); Carter & Chang (2000); Chan et al. (2009); Corà et al. (2012); Delalibera et al. (2015); Dipio et al. (2022); Fasse et al. (2015); Fisher et al. (2014); Govina et al. (2019); Haley et al. (2003); Kim et al. (2017); Jo et al. (2007); Nipp et al. (2016); Olagunju et al. (2016); Rabkin et al. (2009); Rhondali et al. (2015); Sherif et al. (2001); Siminoff et al. (2010); Smith et al. (2003); Valeberg & Grov (2013);
**Sadness**	Jo et al. (2007); Mystakidou et al. (2007); Olagunju et al. (2016);
**Suicidal ideation**	Block (2001); Fisher et al. (2014); Rhondali et al. (2015); Sherif et al. (2001);
**Worthlessness**	Block (2001); Rhondali et al. (2015);
**Outcomes**	**References**
**Decreased well-being**	Bekelman et al. (2008); Nipp et al. (2016); Rhondali et al. (2015);
**Decreased global health status**	Carter & Chang (2000); Haley et al. (2003); Rhondali et al. (2015); Smith et al. (2003); Valeberg & Grov (2013);
**Decreased life satisfaction**	Cheng et al. (2009); Haley et al. (2003); Siminoff et al. (2010);
**Impaired quality of life**	Azevedo et al. (2017); Carter & Chang (2000); Mannarino (2020); Abreu & Júnior (2018); Fasse et al. (2015); Khalil et al. (2021); Kim et al. (2017); Macchi et al. (2020); Collins et al. (2019); Rhondali et al. (2015); Shaffer et al. (2017); Siminoff et al. (2010); Smith et al. (2003); Valeberg & Grov (2013); Wasner et al. (2013);
**Increased mortality**	Shaffer et al. (2017);
**Lack of treatment adherence**	Shaffer et al. (2017).

References

1. Abreu, A. I.; Junior, A. L. Family caregiver workload with the oncologic patient and nursing. *Rev Enfer UFPE on line* **2018**, *12*, 976-86. https://doi.org/10.1097/NCC.0b013e31820d0c23
2. Azevedo, C.; Pessalacia, J. D. R.; Mata, L. R. F. da; Zoboli, E. L. C. P; Pereira, M. da G. Interface between social support, quality of life and depression in users eligible for palliative care. *Rev Esc Enferm USP* **2017**, *51*, e03245. https://doi.org/10.1590/S1980-220X2016038003245
3. Bekelman, D.B.; Hutt, E.; Masoudi, F.A.; Kutner, J.S.; Rumsfeld, J.S. Defining the role of palliative care in older adults with heart failure. *Int J Cardiol* **2008**, *125*, 183-190. https://doi.org/10.1016/j.ijcard.2007.10.005
4. Block, S. D. Perspectives on care at the close of life. Psychological considerations, growth, and transcendence at the end of life: The art of the possible. *JAMA* **2001**, *285*, 2898–2905. https://doi.org/10.1001/jama.285.22.2898.
5. Carter, P. A.; Chang, B. L. Sleep and depression in cancer caregivers. *Cancer Nurs* **2000**, *23*, 410–415. https://doi.org/10.1097/00002820-200012000-00002
6. Chan, W. C. H.; Epstein, I.; Reese, D.; Chan, C. L. W. Family predictors of psychosocial outcomes among Hong Kong Chinese cancer patients in palliative care: living and dying with the "support paradox." *Social Work in Health Care* **2009**, *48*, 519–532. https://doi.org/10.1080/00981380902765824

7. Cheng, W. C.; Schuckers, P. L.; Hauser, G.; Burch, J.; Emmett, J. G.; Walker, B.; Law, E.; Wakefield, D.; Boyle, D.; Lee, M. Psychosocial needs of family caregivers of terminally ill patients. *Psychol Rep* **1994**, *75*, 1243–1250. https://doi.org/10.2466/pr0.1994.75.3.1243
8. Collins, S.; Adile, C.; Ferrera, P.; Cortegiani, A.; Casuccio, A. Symptom hyper-expression in advanced cancer patients with anxiety and depression admitted to an acute supportive/palliative care unit. *Support Care Cancer* **2019**, *27*, 3081-3088. https://doi.org/10.1007/s00520-018-4624-0
9. Corà, A.; Partinico, M.; Munafò, M.; Palomba, D. (2012). Health risk factors in caregivers of terminal cancer patients: a pilot study. *Cancer Nurs*, *35*(1), 38–47. https://doi.org/10.1097/NCC.0b013e31820d0c23
10. Delalibera, M.; Barbosa, A.; Leal, I. Circumstances and consequences of care: characterization of the family caregiver in palliative care. *Cien Saude Colet* **2018**, *23*, 1105–1117. https://doi.org/10.1590/1413-81232018234.12902016
11. Delalibera, M.; Presa, J.; Barbosa, A.; Leal, I. Burden of caregiving and its repercussions on caregivers of end-of-life patients: a systematic review of the literature. *Cien Saude Colet* **2015**, *20*, 2731–2747. https://doi.org/10.1590/1413-81232015209.09562014
12. Dipio, R.; Acuda, W.; Namisango, E.; Nalubega-Mbowa, M. G. Prevalence and factors associated with depressive symptoms among family caregivers of palliative care patients at Hospice Africa Uganda. *Palliat Support Care* **2022**, 20, 375–382. https://doi.org/10.1017/S1478951521000730
13. Fasse, L.; Flahault, C.; Brédart, A.; Dolbeault, S.; Sultan, S. Describing and understanding depression in spouses of cancer patients in palliative phase. *Psycho-Oncology* **2015**, *24*, 1131–1137. https://doi.org/10.1002/pon.3777
14. Fisher, K. A.; Seow, H.; Brazil, K.; Freeman, S.; Smith, T. F.; Guthrie, D. M. Prevalence and risk factors of depressive symptoms in a Canadian palliative home care population: a cross-sectional study. *BMC Palliat Care* **2014**, *13*, 10. https://doi.org/10.1186/1472-684X-13-10
15. Gonzalez, C. P.; Roman-Calderón, J. P.; Limonero, J. T. The relationship between positive aspects of caring, anxiety and depression in the caregivers of cancer patients: The mediational role of burden. *Eur J Cancer Care* **2021**, *30*(1), e13346. https://doi.org/10.1111/ecc.13346
16. Govina, O.; Vlachou, E.; Kalemikerakis, I.; Papageorgiou, D.; Kavga, A.; Konstantinidis, T. Factors associated with anxiety and depression among family caregivers of patients undergoing palliative radiotherapy. *APJON* **2019**, *6*, 283–291. https://doi.org/10.4103/apjon.apjon_74_18
17. Haley, W. E.; LaMonde, L. A.; Han, B.; Burton, A. M.; Schonwetter, R. Predictors of depression and life satisfaction among spousal caregivers in hospice: application of a stress process model. *J Palliat Med* **2003**, *6*, 215–224. https://doi.org/10.1089/109662103764978461
18. Hatano, Y.; Morita, T.; Mori, M.; Aoyama, M.; Yoshida, S.; Amano, K.; Terabayashi, T.; Oya, K.; Tsukuura, H.; Hiratsuka, Y.; Maeda, I.; Kizawa, Y.; Tsuneto, S.; Shima, Y.; Masukawa, K.; Miyashita, M. Association between experiences of advanced cancer patients at the end of life and depression in their bereaved caregivers. *Psycho-Oncology* **2022**, *31*, 1243–1252. https://doi.org/10.1002/pon.5915
19. Hirdes, J. P.; Freeman, S.; Smith, T. F.; Stolee, P. Predictors of caregiver distress among palliative home care clients in Ontario: evidence based on the interRAI Palliative Care. *Palliat Support Care* **2012**, *10*, 155–163. https://doi.org/10.1017/S1478951511000824
20. Jo, S.; Brazil, K.; Lohfeld, L.; Willison, K. Caregiving at the end of life: perspectives from spousal caregivers and care recipients. *Palliat Support Care* **2007**, *5*, 11–17. https://doi.org/10.1017/s1478951507070034
21. Khalil, A.; Khalifeh, A. H.; Al-Rawashdeh, S.; Darawad, M.; Abed. M. Depressive symptoms, anxiety, and quality of life in hemodialysis patients and their caregivers: A dyadic analysis. *J Psycho Res* **2022**, *64*, 426-436. https://doi.org/10.1111/jpr.12339
22. Kim, H.M.; Koh, S. J. ; Hwang, I. C.; Choi, Y. S.; Hwang, S. W.; Lee, Y. J.; Kim, Y. S. Symptom features of terminally ill cancer patients and depression of family caregivers. *Korean J Hospice Palliat Care* **2017**, *20*, 188–193. https://doi.org/10.14475/kjhpc.2017.20.3.188
23. Kochuvilayil, A.; Varma, R. P. Factors associated with screening positive for depression among women caregivers of primary palliative care patients in kerala, india. *J Palliat Med* **2022**, *37*, 510–517. https://doi.org/10.1177/08258597211069210
24. Lai, C.; Luciani, M.; Di Mario, C.; Galli, F.; Morelli, E.; Ginobbi, P.; Aceto, P.; Lombardo, L. Psychological impairments burden and spirituality in caregivers of terminally ill cancer patients. *Eur J Cancer Care* **2018**, *27*. https://doi.org/10.1111/ecc.12674
25. Macchi, Z.A.; Koljack, C. E.; Miyasaki, J. M.; Katz, M.; Galifianakis, N.; Prizer, L.P.; Sillau, S. H.; Kluger, B. M. Patient and caregiver characteristics associated with caregiver burden in Parkinson's disease: A palliative care approach. *Ann Palliat Med* **2019**, *9*, S24-S33. https://doi.org/10.21037/apm.2019.10.01

26. Mystakidou, K.; Tsilika, E.; Parpa, E.; Galanos, A.; Vlahos L. Caregivers of advanced cancer patients: feelings of hopelessness and depression. *Cancer Nurs* **2007**, *30*, 412–418. https://doi.org/10.1097/01.ncc.0000290807.84076.73
27. Nipp, R.D.; El-Jawahri, A.; Fishbein, J.N.; Gallagher, E.R.; Stagl, J.M.; Park, E.R.; Jackson, V.A.; Pirl, W.F.; Greer, J.A.; Temel J.S. Factors associated with depression and anxiety symptoms in family caregivers of patients with incurable cancer. *Annals of Oncology* **2016**, *27*, 1607–1612. https://doi.org/10.1093/annonc/mdw205
28. Olagunju, A. T.; Sarimiye, F. O.; Olagunju, T. O.; Habeebu, M. Y. M.; Aina, O. F. Child's symptom burden and depressive symptoms among caregivers of children with cancers: an argument for early integration of pediatric palliative care. *Ann Palliat Med* **2016**, *5*, 157–165. https://doi.org/10.21037/apm.2016.04.03
29. Özdemir, F.; Doğan, S.; Timuçin Atayoğlu, A. Psychosocial problems of family caregivers of palliative care patients and their spiritual coping styles. *PPC* **2020**, *56*, 636–641. https://doi.org/10.1111/ppc.12479
30. Rabkin, J. G.; McElhiney, M.; Moran, P.; Acree, M.; Folkman, S. Depression, distress and positive mood in late-stage cancer: A longitudinal study. *Psycho-Oncology* **2009**, *18*, 79–86. https://doi.org/10.1002/pon.1386
31. Rhondali, W.; Chirac, A.; Laurent, A.; Terra, J.L.; Filbet, M. Family caregivers' perceptions of depression in patients with advanced cancer: A qualitative study. *Palliat Support Care* **2015**, *13*, 443–450. https://doi.org/10.1017/S1478951513001223
32. Shaffer, K.M.; Jacobs, J.M.; Coleman, J.N.; Temel, J.S.; Rosand, J.; Greer, J.A.; Vranceanu, A.M. Anxiety and depressive symptoms among two seriously medically ill populations and their family caregivers: A comparison and clinical implications. *Neurocritical Care* **2017**, *27*, 180–186. https://doi.org/10.1007/s12028-016-0358-3
33. Sherif, T.; Jehani, T.; Saadani, M., Andejani, A.W. Adult oncology and chronically ill patients: comparison of depression, anxiety and caregivers' quality of life. *EMHJ* **2001**, *7*, 502–509.
34. Siminoff, L. A.; Wilson-Genderson, M; Baker, S., Jr. Depressive symptoms in lung cancer patients and their family caregivers and the influence of family environment. *Psycho-Oncology* **2010**, *19*, 1285–1293. https://doi.org/10.1002/pon.1696
35. Smith, E.M.; Gomm, S.A.; Dickens, C.M. Assessing the independent contribution to quality of life from anxiety and depression in patients with advanced cancer. *Palliat Med* **2003**, *17*, 509–513. https://doi.org/10.1191/0269216303pm781oa
36. Tay, D. L.; Iacob, E.; Reblin, M.; Cloyes, K. G.; Jones, M.; Hebdon, M. C. T.; Mooney, K.; Beck, A. C.; Ellington, L. What contextual factors account for anxiety and depressed mood in hospice family caregivers? *Psycho-Oncology* **2022**, *31*, 316–325. https://doi.org/10.1002/pon.5816
37. Tang, S. T.; Chang, W.C.; Chen, J.S.; Wang, H.M.; Shen, W. C.; Li, C.Y.; & Liao, Y.C. Course and predictors of depressive symptoms among family caregivers of terminally ill cancer patients until their death. *Psycho-Oncology* **2013**, *22*, 1312–1318. https://doi.org/10.1002/pon.3141
38. Tang, S. T.; Li, C. Y.; Liao, Y. C. Factors associated with depressive distress among Taiwanese family caregivers of cancer patients at the end of life. *Palliat Med* **2007**, *21*, 249-257. https://doi.org/10.1177/0269216307077334
39. Tanriverdi, O.; Yavuzsen, T.; Turhal, S.; Kilic, D.; Yalcin, S.; Ozkan, A.; Uzunoglu, S.; Uysal, S. O.; Akman, T.; Aktas, B.; Ulger, S.; Babacan, T.; Komurcu, S.; Yaren, A. ; Cay, S. F. Depression and socio-economical burden are more common in primary caregivers of patients who are not aware of their cancer: TURQUOISE Study by the Palliative Care Working Committee of the Turkish Oncology Group (TOG). *Eur J Cancer Care* **2016**, *25*, 502–515. https://doi.org/10.1111/ecc.12315
40. Valeberg, B. T.; Grov, E. K. Symptoms in the cancer patient – Of importance for their caregivers' quality of life and mental health?. *EJON* **2013**, *17*(1), 46–51. https://doi.org/10.1016/j.ejon.2012.01.009
41. Wasner, M.; Paal, P.; & Borasio, G. D. Psychosocial care for the caregivers of primary malignant brain tumor patients. *J Soc Work End-Life* **2013**, *9*(1), 74–95. https://doi.org/10.1080/15524256.2012.758605
